# Semi-quantitative measurements of chemokine receptor 4-targeted ^68^Ga-pentixafor PET/CT in response assessment of Waldenström macroglobulinemia/lymphoplasmacytic lymphoma

**DOI:** 10.1186/s13550-021-00852-0

**Published:** 2021-10-29

**Authors:** Qingqing Pan, Xinxin Cao, Yaping Luo, Jian Li, Fang Li

**Affiliations:** 1 Department of Nuclear Medicine, Chinese Academy of Medical Sciences and Peking Union Medical College Hospital, Wangfujing, Dongcheng District, Beijing, 100730 People’s Republic of China; 2 Beijing Key Laboratory of Molecular Targeted Diagnosis and Therapy in Nuclear Medicine, Beijing, People’s Republic of China; 3 Department of Hematology, Chinese Academy of Medical Sciences and Peking Union Medical College Hospital, Wangfujing, Dongcheng District, Beijing, 100730 People’s Republic of China

**Keywords:** Waldenström macroglobulinemia, CXCR4, ^68^Ga-pentixafor, PET/CT, Response assessment

## Abstract

**Purpose:**

^68^Ga-pentixafor PET/CT was reported to have a high sensitivity in detecting tumor involvement of Waldenström macroglobulinemia/lymphoplasmacytic lymphoma (WM/LPL) in our previous study. We aimed to further investigate the semi-quantitative measurements of ^68^Ga-pentixafor PET/CT in response assessment in WM/LPL.

**Methods:**

Fifteen patients with WM/LPL were recruited in a prospective cohort study and underwent both ^68^Ga-pentixafor and ^18^F-FDG PET/CT at baseline and post-treatment. PET/CT-based responses were analyzed with semi-quantitative assessments of metabolic tumor volume (MTV) and total lesions glycolysis/uptake (TLG_FDG_ and TLU_CXCR4_), and the correlation between PET/CT-based response and clinical response, monoclonal protein and IgM response was analyzed.

**Results:**

After chemotherapy, 5 patients had complete response or very good partial response, 8 had partial response or minimal response and 2 had progressive disease. In quantitative analysis, ^68^Ga-pentixafor PET/CT-based response (measured in ∆TLU_CXCR4_%, ∆MTV_CXCR4_%, ∆SUVpeak%) showed a significant direct correlation with clinical response, monoclonal protein and IgM response (*p* < 0.01). However, ^18^F-FDG PET/CT-based response was independent from clinical response (*p* > 0.05).

**Conclusions:**

The semi-quantitative measurements of ^68^Ga-pentixafor PET/CT outperformed ^18^F-FDG PET/CT in response assessment of WM/LPL.

**Supplementary Information:**

The online version contains supplementary material available at 10.1186/s13550-021-00852-0.

Waldenström macroglobulinemia/lymphoplasmacytic lymphoma (WM/LPL) is a lymphoplasmacytic lymphoma in which the bone marrow is infiltrated by immunoglobulin (Ig)M-producing clonal lymphoplasmacytic cells. Similar to other indolent lymphomas, treatment is indicated for patients with symptomatic WM/LPL, for example, systemic symptoms, systemic or bulky lymphadenopathy, anemia or thrombocytopenia, hyperviscosity, secondary amyloidosis, paraneoplastic neuropathy, etc. [1]. As for assessing treatment response of WM/LPL, consensus-based uniform response criteria were proposed by the International Working Group on WM in 2002 and were then revised in the subsequent International Workshops [2]. These uniform response criteria are based mainly on the change of serum monoclonal IgM protein level after treatment, since IgM serves as a good marker for tracking disease burden in a particular patient with WM/LPL [3, 4]. If there is significant lymphadenopathy or hepatosplenomegaly at baseline, a CT scan of the chest/abdomen is needed to determine the resolution of extramedullary disease.

^18^F-FDG PET/CT has been widely used for staging and response assessment of FDG-avid nodal lymphomas. The International Working Group criteria for reviewing post-treatment ^18^F-FDG PET scans are based on visual interpretation using the 5-point Deauville scale with mediastinal blood pool and liver as the comparator [5]. However, ^18^F-FDG PET/CT is not recommended in WM/LPL neither for staging or treatment response, as this indolent lymphoma is usually not FDG-avid [6], and classification of diffuse bone marrow involvement of lymphoma (which is the characteristic of WM/LPL) is difficult with ^18^F-FDG PET/CT. A study on the role of ^18^F-FDG PET/CT in WM/LPL showed a sensitivity of only 43% in detection of bone marrow involvement; moreover, this study found there was no statistical correlation between ^18^F-FDG PET/CT response and monoclonal protein response [7].

As chemokine receptor 4 (CXCR4), a key factor for tumor growth and metastasis, is overexpressed in the malignant B-cells of patients with WM/LPL at a high level [8–10], we previously conducted a prospective cohort study and reported that ^68^Ga-pentixafor, a CXCR4-targeted PET probe, was obviously more sensitive than ^18^F-FDG in detecting tumor involvement of WM/LPL (100% vs. 58.8%) [11–13]. We also found ^68^Ga-pentixafor is superior to ^18^F-FDG in determining disease response and residual disease after treatment [11, 14]. Recently, we reported the results of visual response assessment of ^68^Ga-pentixafor and ^18^F-FDG PET/CT based on 5-point scale in 15 patients with WM/LPL, and found that ^68^Ga-pentixafor PET-based response was consistent with clinical response categories, but ^18^F-FDG PET/CT missed the response in nearly half of the patients [15]. Based on the above evidence, we intend to further investigate if the quantitative tumor burden measurements on ^68^Ga-pentixafor PET/CT can be a precise and objective biomarker to assess the treatment response of WM/LPL, and ^18^F-FDG PET/CT was also analyzed as a reference.

## Methods

### Study design and patients

This is a retrospective analysis of the data from our prospective cohort study on the role of ^68^Ga-pentixafor PET/CT in WM/LPL approved by the Institutional Review Board of Peking Union Medical College Hospital (protocol ZS-1113) and registered at ClinicalTrials.gov (NCT 03436342). A total of 15 patients with newly diagnosed symptomatic WM/LPL at the Department of Hematology, Peking Union Medical College Hospital, were consecutively recruited from March 2018 to June 2020. Written informed consent was obtained from each patient. Laboratory tests and bone marrow evaluation for WM/LPL were done at enrollment. Patients were then referred for ^18^F-FDG and ^68^Ga-pentixafor PET/CT for baseline evaluation that were performed within 1 week after enrollment. Chemotherapy against WM/LPL was started within 2 weeks thereafter. After completion of chemotherapy, all the patients underwent follow-up ^18^F-FDG and ^68^Ga-pentixafor PET/CT. In the meantime, clinical response was evaluated according to the consensus response criteria on WM/LPL [3]. The response category was classified as complete response (CR), very good partial response (VGPR), partial response (PR), minimal response (MR), stable disease (SD), and progressive disease (PD) mainly based on monoclonal IgM protein level: CR is defined as absence of serum monoclonal IgM protein by immunofixation; VGPR is monoclonal IgM protein detectable ≥ 90% reduction in serum IgM level from baseline; PR is defined as monoclonal IgM protein detectable ≥ 50% but < 90% reduction in serum IgM level from baseline; MR means monoclonal IgM protein detectable ≥ 25% but < 50% reduction in serum IgM level from baseline; SD is monoclonal IgM protein detectable < 25% reduction and < 25% increase in serum IgM level from baseline; PD is defined as ≥ 25% increase in serum IgM level from lowest nadir.

### PET/CT study

The DOTA-CPCR4-2 peptide was purchased from CSBio Co (CA 94025, USA). The radiolabeling of ^68^Ga-pentixafor was performed manually before injection according to the procedures as previously published. ^18^F-FDG was synthesized in house with an 11 MeV cyclotron (CTI RDS 111, Siemens, Germany). The PET scans were performed with dedicated PET/CT scanners (Biograph 64 Truepoint TrueV, Siemens, Germany; Polestar m660, SinoUnion, China) from the tip of the skull to the middle thigh. For ^18^F-FDG PET/CT, the patients fasted for at least 6 h, and the blood glucose levels were monitored (4.7–6.9 mmol/L) before an injection of ^18^F-FDG (5.55 MBq/kg). The PET/CT images (2 min/bed) were acquired with an uptake time of 75.0 ± 13.2 (mean ± SD) min. For ^68^Ga-pentixafor PET/CT, imaging was performed (2–4 min/bed) with an uptake time of 45.9 ± 19.7 min after an injection of 85.1 ± 27.4 MBq of ^68^Ga-pentixafor. The acquired data were reconstructed using the ordered-subset expectation maximization method (Biograph 64: 2 iterations, 8 subsets, Gaussian filter, image size of 168 × 168; Polestar m660: 2 iterations, 10 subsets, Gaussian filter, image size of 192 × 192).

### Imaging analysis

Two experienced nuclear medicine physicians (YL and QP) visually assessed the PET/CT images and were in consensus for image interpretation. The presence and sites of tumor involvements, and the intensity of the uptake in the lesions were recorded. Semi-quantitative measurements of whole-body tumor burden were done in ^68^Ga-pentixafor and ^18^F-FDG PET/CT both at baseline and follow-up, measured as metabolic tumor volume (MTV, defined as the sum of the metabolic volumes of all tumors) and total lesions glycolysis/uptake (TLG_FDG_ and TLU_CXCR4_, defined as the sum of individual MTV multiplied by its mean SUV) [16, 17]. PET/CT data were transferred in DICOM format to MIM workstation (version 6.6.11, MIM Software, USA). Then, a rectangular volume of interest was drawn including bone marrow and all focal lesions previously localized in the PET/CT images. Subsequently, tumor contours were first semiautomatically segmented with a SUV cutoff of 2.5. The contours were then checked and manually adjusted to exclude the physiological uptakes in heart, urinary tract, brain, vocal cords, liver, etc. Afterward, volumetric parameters of MTV, TLG _FDG_/TLU_CXCR4,_ and SUVpeak were automatically obtained from the statistics generated with the final volumetric extraction.

### Statistical analysis

The percentage of change in semi-quantitative PET/CT parameters between the baseline PET/CT (*U*_pre_) and post-treatment PET/CT (*U*_post_) was described by ∆*U*% (∆*U*% = (*U*_pre_ − *U*_post_)/*U*_pre_ × 100%, *U* referred to TLG_FDG_/TLU_CXCR4_, MTV and SUVpeak). The percentage of change in serum monoclonal protein (M-protein) and total serum IgM level between the baseline and the time of follow-up PET/CT was recorded as ∆M-pro% and ∆IgM%, respectively. The clinical response categories of CR, VGPR, PR, MR, SD and PD were assigned as score 1–6, respectively, for correlation analysis. The correlations between ∆*U*% and clinical response, ∆M-pro%, ∆IgM% were analyzed by Spearman’s rank correlation coefficients (for skewed data). A *p* value < 0.05 was considered statistically significant. Statistical analyses were done with the MedCalc software (version 19.6.4).

## Results

### Clinical characteristics

Fifteen patients with newly diagnosed symptomatic WM/LPL (12 men and 3 women; 60.9 ± 8.6 [range 48–76] y old) were analyzed in the present study. The median level of M-protein in recruited patients was 23.7 g/L (range 2.1–62.8 g/L), and the median level of β2-microglobulin was 5.5 mg/L (range 2.9–12.6 mg/L). According to the International Scoring System for WM (ISS-WM) [18], 3 patients were classified as being at high risk, 10 patients were classified as being at intermediate risk and one patient was classified as being at low risk. One patient with IgD LPL (patient 6) had unknown risk stratification because the serum M-protein and β2-microglobulin levels were not measured. Mutation of myeloid differentiation primary response 88, which has been identified in more than 90% of WM/LPL patients [19], was identified in all patients in the present study. Four patients (26.7%) were found to have a CXCR4 mutation.

All patients had bone marrow involvement confirmed by bone marrow aspiration and biopsy. Bone marrow involvement of WM/LPL showed markedly increased uptake of ^68^Ga-pentixafor (SUVmax 7.9 ± 2.5, range 5.1–14.8), but was less FDG-avid (SUVmax 3.4 ± 0.9, range 2.1–5.3). Besides bone marrow, WM/LPL involvement was seen in lymph nodes (12/15 patients, including cervical, subclavian, axillary, mediastinal, hepatic/splenic hila, peri-pancreas, para-aortic, bilateral iliac and inguinal nodes; short axis diameter of the largest lymph node, 20.8 ± 9.2 mm, range 11–36 mm), paramedullary space (3/15 patients, including soft tissues around the sternum, thoracic and lumbar vertebrae, and presacral space), liver (2/15), pancreas (1/15), and spleen (2/15). The median TLU_CXCR4_ and MTV_CXCR4_ of ^68^Ga-pentixafor PET/CT at baseline were 4036.3 (mean ± SD 5891.6 ± 5374.6; range 273.0–22,032.3) and 1189.1 (mean ± SD 1528.7 ± 1129.4; range 94.3–4480.8), respectively; and the median TLG_FDG_ and TMV_FDG_ were 672.9 (mean ± SD 672.9 ± 892.1; range 0–2902.6) and 232.4 (mean ± SD 232.4 ± 302.9; range 0–983.3), respectively. The baseline clinical characteristics and tumor involvement are summarized in Table 1.

**Table 1:** Patients’ clinical characteristics and treatment response

Patient	Age/sex	ISS-WM*	M-protein type	IgM (g/L)	M-protein (g/L)	β2-MG (mg/L)	Involvement at baseline	Chemotherapy regimens^a^ (cycles)	Clinical response^†^
1	61/M	Intermediate	IgMκ	30.49	18.5	6.13	BM, LN, PMD, nerve root	R-FC(5) + R-DC(1)	CR
2	72/M	High	IgMκ	5.78	2.1	8.93	BM, LN	DRC(7)	PR
3	72/M	Intermediate	IgMλ	15.2	10.5	3.27	BM	DRC(6)	VGPR
4	64/M	Intermediate	IgMλ	23.69	10.6	5.71	BM, LN	BRD(6)	CR
5	64/M	Intermediate	IgMκ	53.3	32.5	5.27	BM, LN	DRC(8)	VGPR
6	48/F	N/A	IgDκ	6.67(IgD)^‡^	6.67	N/A	BM, LN	DRC(6)	PR
7	55/F	Low	IgMκ	82.49	35.6	2.93	BM, LN	DRC(1) + BRD(4) + BD(1)	PR
8	52/F	Intermediate	IgMκ	38.13	21.6	3.34	BM, spleen	DRC(6)	PR
9	58/M	Intermediate	IgMκ	43.48	27.9	12.6	BM, LN, liver, pancreas, PMD	R2(5)	PR
10	48/M	Intermediate	IgMλ	50.9	32.5	6	BM, LN, liver, PMD	DRC(6)	PD
11	62/M	High	IgMκ	33.42	23.7	6	BM, LN	DRC(4)	VGPR
12	76/M	High	IgMλ	96.02	62.8	3.6	BM, LN	BRD(8)	PR
13	53/M	Intermediate	IgMλ	79.89	56.5	3.5	BM, LN, spleen	Chlorambucil(2) + BCD(5)	MR
14	64/M	Intermediate	IgMλ	52.73	30.6	5.9	BM, LN	BRD(4) + DRC(2)	PR
15	64/M	Intermediate	IgMκ	7.57	3.6	3.5	BM	Chlorambucil(5)	PD

*β2-MG* β2-microglobulin, *BM* bone marrow, *LN* lymph node, *PMD* paramedullary disease

*International Staging System for WM (ISS-WM) prognostic scoring includes age of > 65 y, ß2-microglobulin level of > 3 mg/L, hemoglobin level of ≤ 11.5 g/dL, platelet count of ≤ 100 × 10^9^/L, and IgM level of > 7 g/dL

^‡^Serum IgD level was measured as IgD-type M-protein level

^†^*CR* complete response, *VGPR* very good partial response, *PR* partial response, *MR* minimal response, *SD* stable disease, *PD* progressive disease

^a^*R* rituximab, *D* dexamethasone, *C* cyclophosphamide, *B* bortezomib, *F* fludarabine, *R2* lenalidomide plus rituximab

### Quantitative response assessment with PET/CT after chemotherapy

**Fig. 1:**
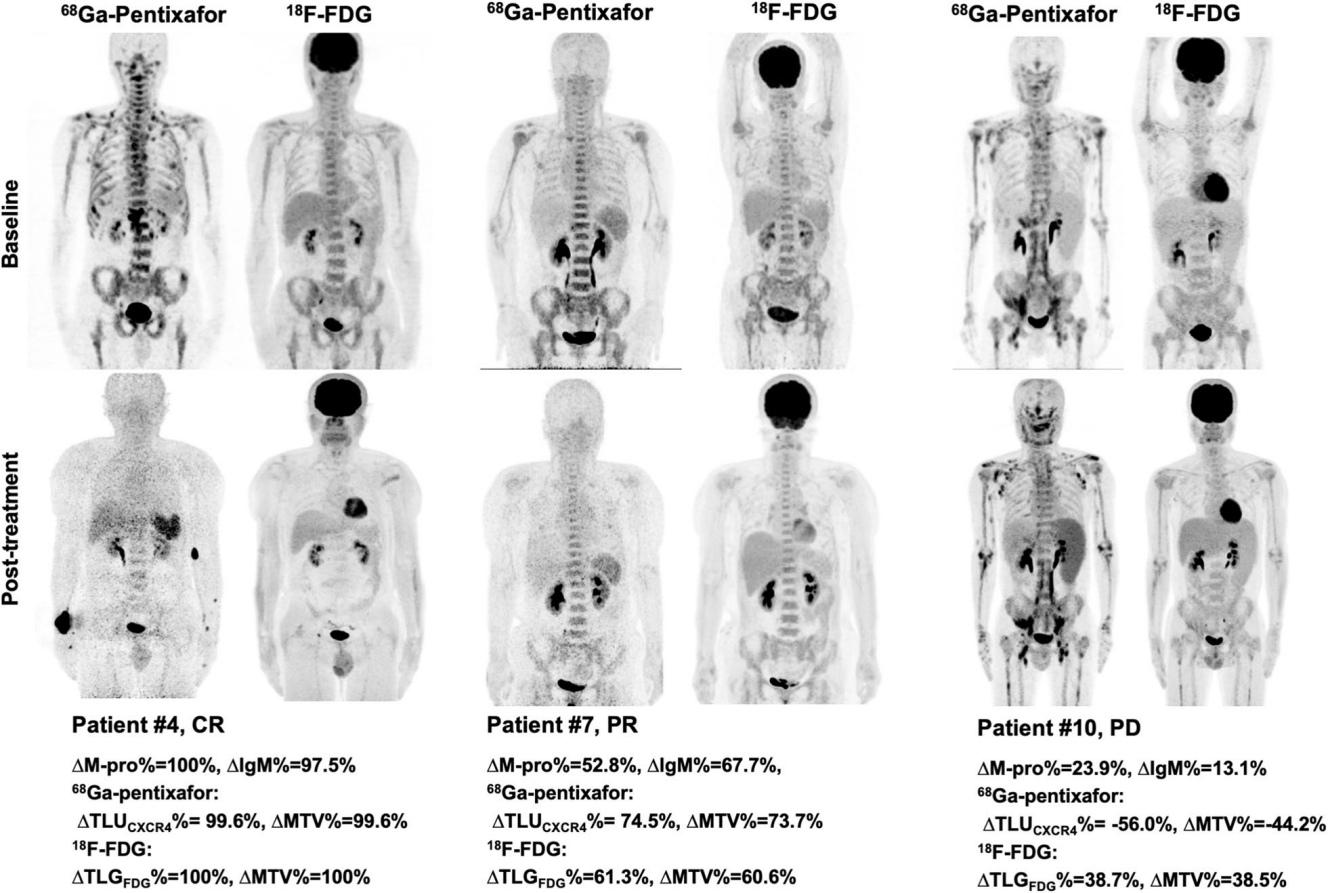
Examples of 3 patients with clinical CR, PR, and PD after chemotherapy showing different response in ^68^Ga-pentixafor and ^18^F-FDG PET/CT. *CR* complete response, *PR* partial response, *PD* progressive disease

After baseline PET/CT, all of the 15 patients received chemotherapy, and follow-up PET/CT was performed after completion of treatment. The intervals between the last cycle of chemotherapy and the follow-up PET/CT were 2 weeks to 10 months (median 7 weeks). According to the consensus response criteria for WM/LPL [3], 5 patients achieved CR or VGPR after chemotherapy with a reduction of IgM and M-protein greater than 90% (range 90.4 to 100%); specifically, the 2 patients with CR also had a complete resolution of the lymph node involvement and other extramedullary disease (short axis diameter of the largest lymph node at baseline, 31 mm and 26 mm, respectively). Eight patients were evaluated as PR or MR with a reduction of IgM and M-protein less than 90% (range 32.1–85.7%), and 2 patients had PD with an increase in IgM and M-protein from the lowest nadir at follow-up (example in Fig. 1).

All of the 5 patients who achieved CR or VGPR after chemotherapy showed a consistent reduction of tumor burden measured in ^68^Ga-pentixafor PET/CT (median ∆TLU_CXCR4_%, 99.6%, mean ± SD 98.4% ± 2.5%, range 93.5–100%; median ∆MTV_CXCR4_%, 99.6%, mean ± SD 97.7% ± 3.8%, range 90.0–100%). Complete normalization of bone marrow uptake of ^68^Ga-pentixafor and complete resolution of extramedullary disease was also noted in 4 patients. The remaining one patient with clinical VGPR showed significantly reduced uptake of ^68^Ga-pentixafor in bone marrow, but residual lymph node disease avid for ^68^Ga-pentixafor was also noted, although the reduction of TLU_CXCR4_ and MTV_CXCR4_ was consistent with IgM response (∆M-pro% 100%, ∆IgM% 97.3%, ∆TLU_CXCR4_% 93.5%, ∆MTV_CXCR4_% 90.0%).

In the 8 patients with clinical PR or MR after chemotherapy, a significant reduction of TLU_CXCR4_ and MTV_CXCR4_ was noted (median ∆TLU_CXCR4_%, 90.5%, mean ± SD 86.2% ± 11.8%, range 65.8–99.8%; median ∆MTV_CXCR4_%, 89.4%, mean ± SD 85.4% ± 12.4%, range 62.3–99.8%), consistent with partial reduction of lesion uptake. In the 2 patients with clinical progression, one patient showed consistent increase in tumor burden measured in ^68^Ga-pentixafor PET/CT (∆TLU_CXCR4_%, − 56.0%; ∆MTV_CXCR4_%, − 44.2%). The other patient had a reduction of both TLU_CXCR4_ and MTV_CXCR4_ at follow-up (∆TLU_CXCR4_%, 48.4%; ∆MTV_CXCR4_%, 44.6%), but new lesions in the lymph nodes were detected in bilateral iliac regions.

In correlation analysis, ∆TLU_CXCR4_%, ∆MTV_CXCR4_% and ∆SUVpeak% in ^68^Ga-pentixafor PET/CT showed a significant direct correlation with clinical response, ∆M-pro%, and ∆IgM% values (*p* < 0.01, Table 2). However for ^18^F-FDG PET/CT, the uptake values (∆TLG_FDG_%, ∆MTV_FDG_%, and ∆SUVpeak%) were independent from clinical response, ∆M-pro%, and ∆IgM% (*p* > 0.05, Table 2). Semi-quantitative measurements of ^68^Ga-pentixafor and ^18^F-FDG PET/CT at baseline and follow-up were listed patient by patient in Additional file 1: Table S1.

**Table 2:** Spearman’s rank correlation test of semi-quantitative PET/CT-based response and clinical response

	^68^Ga-pentixafor PET/CT			^18^F-FDG PET/CT		
	∆TLU_CXCR4_%	∆MTV_CXCR4_%	∆SUVpeak%	∆TLG_FDG_%	∆MTV_FDG_%	∆SUVpeak%
Clinical response						
*r* (95% CI)	− 0.780 (− 0.923 to − 0.446)	− 0.761 (− 0.916 to − 0.408)	− 0.725 (− 0.902 to − 0.339)	− 0.411 (− 0.763 to 0.129)	− 0.411 (− 0.763 to 0.129)	− 0.357 (− 0.735 to 0.190)
*p*	0.0006*	0.001*	0.0022*	0.1283	0.1283	0.1910
∆M-pro%						
*r* (95% CI)	0.821 (0.532 to 0.938)	0.796 (0.478 to 0.929)	0.724 (0.337 to 0.902)	0.439 (− 0.0942 to 0.777)	0.439 (− 0.0942 to 0.777)	0.447 (− 0.0846 to 0.781)
*p*	0.0002*	0.0004*	0.0023*	0.1013	0.1013	0.0948
∆IgM%						
*r* (95% CI)	0.764 (0.414 to 0.917)	0.739 (0.365 to 0.908)	0.654 (0.212 to 0.873)	0.469 (− 0.0573 to 0.791)	0.469 (− 0.0573 to 0.791)	0.430 (− 0.106 to 0.772)
*p*	0.0009*	0.0016*	0.0082*	0.0780	0.0780	0.1097

*The correlation coefficient is significant

## Discussion

Our study determined that ^68^Ga-pentixafor PET/CT-based response, measured as ∆TLU_CXCR4_%, ∆MTV_CXCR4_%, ∆SUVpeak%, was well correlated with the clinical response and M-protein response in WM/LPL after chemotherapy. However ^18^F-FDG PET/CT-based response was independent from the clinical response. Therefore, it is suggested that the semi-quantitative measurements of whole-body tumor burden in ^68^Ga-pentixafor PET/CT might serve as a good biomarker for tracking disease burden in a particular patient with WM/LPL and for assessing treatment response.

Accurate discerning the depth of treatment response is important for stratifying patients and predicting outcomes. In WM/LPL, serum IgM M-protein levels is used as a determinant of disease response to therapy and to follow disease burden for an individual patient [2]. In our study, the 5 patients with CR or VGPR after chemotherapy showed a consistent reduction of tumor burden measured in ^68^Ga-pentixafor PET/CT (median ∆TLU_CXCR4_%, 99.6%, mean ± SD 98.4% ± 2.5%, range 93.5–100%); however, we noticed that ^68^Ga-pentixafor PET/CT did not distinguish VGPR from CR in 2 patients (^68^Ga-pentixafor PET/CT presented with a reduction over 99% of TLU_CXCR4_ and MTV_CXCR4_ from baseline in 4 patients who achieved CR or VGPR). Considering there was no difference in prognosis between patients with CR and with VGPR [20], we think it may not affect our further studies in spite of the incapability of ^68^Ga-pentixafor PET/CT to distinguish VGPR from CR.

For the determination of CR or VGPR, increased stringency is required as patients achieving VGPR or better response usually have improved progression free survival [20–22]. In addition to IgM response, CR or VGPR requires complete resolution of extramedullary disease (e.g., lymphadenopathy, splenomegaly) if present at baseline [3]. In the 4 patients with CR or VGPR and with a reduction over 99% of TLU_CXCR4_ and MTV_CXCR4_, there was complete normalization of bone marrow uptake of ^68^Ga-pentixafor and complete resolution of extramedullary disease. Interestingly, in the remaining one patient with clinical VGPR (patient 11, ∆TLU_CXCR4_% 93.5%, ∆MTV_CXCR4_% 90.0%), ^68^Ga-pentixafor PET/CT still detected residual lymph node disease (missed in ^18^F-FDG PET/CT), which was discordant with the stringent response criteria of VGPR, if using ^68^Ga-pentixafor PET/CT as the imaging modality to assess extramedullary disease. Considering such circumstances, we think the stringency for determination of a CR or VGPR state might be improved with the application of ^68^Ga-pentixafor PET/CT. However, it is not clear whether such change of treatment response categories has an impact on patients’ prognosis.

Clinical PD is defined as ≥ 25% increase in serum IgM level from lowest nadir and/or progression in clinical features attributable to the disease. As interim PET/CT was not performed during the course of chemotherapy in our study, the change of TLU_CXCR4_ or MTV _CXCR4_ at follow-up from baseline may possibly underestimate the disease progression. Therefore, visual assessment of ^68^Ga-pentixafor PET/CT is important for accurate interpretation of PD response. For example, in patient 15 with PD after chemotherapy, despite a nearly 50% reduction of TLU_CXCR4_ and MTV _CXCR4_ from baseline, there were new emerging lymph node diseases detected by both ^18^F-FDG and ^68^Ga-pentixafor PET/CT at follow-up, consistent with the clinical response classification of PD.

It is becoming clear that discrepancies can exist between monoclonal IgM protein responses and tumor reduction. For example, IgM responses are typically slow with monoclonal antibody-based therapy, as these agents selectively deplete the CD20+ B-cell component with sparing of the CD138+ plasma cell component [3]; transient increase in monoclonal IgM protein level (IgM flare) can occur following rituximab infusion [23]; in patients treated with bortezomib, tumor reduction in the bone marrow may not be proportional to the suppression of IgM levels [24]. Taking account of these cases, additional investigations are needed to confirm the disease response along with IgM levels, and to help with precise identification of the depth of response and early recognition of disease progression. Further studies are warranted whether ^68^Ga-pentixafor PET/CT could play such a role to supplement the current response criteria.

Our study had several limitations. First, it had a relatively small number of patients. However, we reported this preliminary result and found the possible significance of ^68^Ga-pentixafor PET/CT in response assessment of WM/LPL. Further study with a larger cohort is needed to confirm the results derived from this pilot study and to further evaluate its impact on predicting patients’ outcomes. Second, the time interval between the end of treatment and post-treatment PET/CT was variant, ranging from 2 weeks to 10 months. However, the evaluation of clinical response and laboratory tests were performed in the same period of PET/CT, so the analysis of PET/CT response was not biased. Considering the surface expression of CXCR4 is a dynamic process influenced by concomitant chemotherapy [25, 26], we did not perform interim PET/CT and scheduled the post-treatment PET/CT at least 2 weeks after completion of chemotherapy.

## Conclusions

In the present study, we found that ^68^Ga-pentixafor PET/CT-based quantitative response after chemotherapy was well correlated with clinical response categories and monoclonal IgM protein level, a major determinant for disease response in patients with WM/LPL. Further investigation is warranted to evaluate the value of quantitative ^68^Ga-pentixafor PET/CT in patients’ prognosis and survival in a larger cohort of WM/LPL.

## Supplementary Information

**Additional file 1.** **Supplement Table 1.** The volume measurements patient by patient.

## Data Availability

The datasets generated during and/or analyzed during the current study are available from the corresponding author on reasonable request.
