# Supplementary material for: Semi-quantitative measurements of chemokine receptor 4-targeted 68Ga-pentixafor PET/CT in response assessment of Waldenström macroglobulinemia/lymphoplasmacytic lymphoma
Source: EJNMMI Res. 2021 Oct 29;11:110. doi: 10.1186/s13550-021-00852-0 (PMC8556471; doi:10.1186/s13550-021-00852-0)
Supplement: Supplementary file 1 — Additional file 1. Supplement Table 1. The volume measurements patient by patient. [file 13550_2021_852_MOESM1_ESM.docx]

Supplement Table 1. The volume measurements patient by patient

| Patient | Age/sex | Clinical response | ^68^Ga-pentixafor PET/CT | | | |  | ^18^F-FDG PET/CT | | | |
| --- | --- | --- | --- | --- | --- | --- | --- | --- | --- | --- | --- |
|  |  |  | Pre-treatment | | Post-treatment | |  | Pre-treatment | | Post-treatment | |
|  |  |  | TLU_CXCR4_ | MTV_CXCR4_ | TLU_CXCR4_ | MTV_CXCR4_ |  | TLG_FDG_ | MTV_FDG_ | TLG_FDG_ | MTV_FDG_ |
| 1 | 61/M | CR | 4036.32 | 1189.05 | 0 | 0 |  | 90.25 | 33.29 | 0 | 0 |
| 2 | 72/M | PR | 1025.74 | 346.04 | 58.28 | 20.29 |  | 30.8 | 11.6 | 0 | 0 |
| 3 | 72/M | VGPR | 273 | 94.25 | 1.89 | 0.7 |  | 0 | 0 | 0 | 0 |
| 4 | 64/M | CR | 6747.02 | 1858.1 | 24.94 | 8.36 |  | 260.61 | 96.1 | 0 | 0 |
| 5 | 64/M | VGPR | 4950.63 | 1382.99 | 15.97 | 5.79 |  | 262 | 97.27 | 0 | 0 |
| 6 | 48/F | PR | 1852.94 | 585.36 | 124.9 | 44.93 |  | 396.19 | 146.02 | 0 | 0 |
| 7 | 55/F | PR | 2157.47 | 658.5 | 550.41 | 173.04 |  | 1081.19 | 371.33 | 417.62 | 146.48 |
| 8 | 52/F | PR | 3950.59 | 914.47 | 1351.84 | 344.3 |  | 26.45 | 10.26 | 0 | 0 |
| 9 | 58/M | PR | 9094.98 | 2607.49 | 1113.33 | 353.55 |  | 5.54 | 2.12 | 0 | 0 |
| 10 | 48/M | PD | 3367.76 | 1002.7 | 5252.53 | 1445.74 |  | 52.12 | 19.33 | 31.94 | 11.88 |
| 11 | 62/M | VGPR | 22032.28 | 4480.75 | 1421.42 | 446.51 |  | 1754.43 | 601.91 | 339.83 | 121.05 |
| 12 | 76/M | PR | 3833.87 | 1183.71 | 7.8 | 2.84 |  | 85.07 | 31.6 | 0 | 0 |
| 13 | 53/M | MR | 9109.71 | 2266.62 | 2192.05 | 532.24 |  | 1574.02 | 538.93 | 13.8 | 5.23 |
| 14 | 64/M | PR | 10171.7 | 2661.86 | 159.14 | 56.29 |  | 2902.59 | 983.25 | 1479.76 | 497.61 |
| 15 | 64/M | PD | 5770.36 | 1699.03 | 2979.97 | 940.64 |  | 1571.76 | 542.78 | 1696.4 | 568.51 |
